# Hedonal and Its Limitations

**Published:** 1912-11-09

**Authors:** 


### ROYAL SOCIETY OF MEDICINE.

### Hedonal and its Limitations.

At the meeting of the Section of Anæsthetics on Friday Dr. G. A. H. Barton related a death which occurred after hedonal anæsthesia, and it was made the basis of a discussion on the advantages and limitations of that drug. The unfortunate patient was a tailor who was much reduced in condition owing to participating in the strike, and had fairly advanced pulmonary tuberculosis. Dr. Trevor, who made the post-mortem examination, said the body was that of a spare man, and he found adhesions all over the right lung and at the left apex. Both lungs were riddled with miliary tubercle, and in both there was apical fibro-caseous tubercle, with a fair-sized cavity at the right apex. He attributed death to heart failure while the patient was suffering from the toxœmia of tuberculosis, such death having been hastened by the administration of hedonal, for which evidently he was not a suitable subject, especially as one effect of the drug was to annul the respiratory function. The lungs contained inspired blood and were mottled, presenting the so-called "tigroid" appearance. Dr. Z. Mennell gave his long experience with hedonal, which he had administered 196 times, and there had been no death within twelve hours of the operation. The prolonged sleep of the patient was usually considered beneficial and obviated the need of giving a narcotic after restoring him to bed. The contra-indications were, in his opinion, the following: (1) Any operation which would cause bleeding into the larynx or trachea; (2) cases where the blood pressure was high—these required large quantities of hedonal to induce anæsthesia; (3) cases in which anæsthesia could be obtained as safely and as satisfactorily by other means—*e.g.*, at St. Thomas's Hospital it was not now used as a routine anæsthetic. Among the advantages claimed for hedonal were that the patient could be put into any posture; so long as a free air-way was maintained and the anæsthetist was out of way, there was no vomiting. Out of 196 cases of intracranial surgery hedonal was given in 43, and the mortality rates within forty-eight hours from the operation were: with chloroform, 20 per cent.; ether, 13.6 per cent.; and with hedonal, 3.2 per cent. Most of the cases had chloroform and fewest had ether. The rate of administration which he practised was 100 c.c. every two or three minutes. Cyanosis he regarded as the greatest danger-signal in the case of this drug. Chest disease was not necessarily a contra-indication. The corneal reflex he regarded as valueless; the skin reflex was of more service, and its abolition meant that the patient was too deeply under. In the last forty cases there had been no need to draw the tongue forward as he had been giving a smaller dose of the drug than formerly. In one case, by giving morphia at intervals, as little as 240 c.c. was given. Dr. Silk expressed the opinion that the doses of hedonal given and recommended were too large to be safe. But he thought hedonal might very well be used as an adjunct to other anæsthetics. The advocates of hedonal at first said it was suitable for all cases, but they now admitted that it had its limitations, and that its special aptitude was for cerebral surgery. Dr. J. D. Mortimer regretted that all the deaths from hedonal had not been recorded. Dr. Barton replied on the points raised that possibly, if he could have used some apparatus with a double reservoir, one containing hedonal and the other saline solution, and turned the latter on at the later stages, the result might have been different. There were no symptoms to cause him uneasiness in this case when he left the hospital; they developed later.

